# Comparing health insurance and survey data in estimating prevalence of chronic diseases

**DOI:** 10.1093/eurpub/ckac131.145

**Published:** 2022-10-25

**Authors:** E Mertens, JL Peñalvo, S Vandevijvere

**Affiliations:** 1 Lifestyle and Chronic Diseases, Sciensano, Brussels, Belgium; 2 Non-communicable Diseases, Institute of Tropical Medicine, Antwerp, Belgium

## Abstract

**Background:**

Population prevalence of chronic conditions can be estimated from national health surveys and from administrative data sources such as insurance records. This study evaluated the agreement between the Belgian Health Interview Survey (BHIS) and the Belgian compulsory health insurance data (BCHI) in ascertaining chronic hypertension, hypercholesterolemia and diabetes in Belgium.

**Methods:**

The most recent cycle of BHIS (2018) provided the self-reported prevalence of diabetes, hypertension, and hypercholesterolemia among a representative sample of Belgian adults. For BCHI, the chronic conditions were attributed for every individual in the BHIS reviewing the medication prescription records identified using the ATC/DDD system. These two data sources were linked through unique identifiers by STATBEL. Disease prevalence, measures of agreement, and measures of concordance were estimated. Logistic regression was performed to determine the factors affecting agreement between BHIS and BCHI’s disease classifications.

**Results:**

Data linkage was done for 9,753 individuals aged 15 years and older. From the sample, BHIS and BCHI respectively identified 5.9% and 5.6% diabetes cases, 18% and 24% of hypertension cases, and 18% and 16% of hypercholesterolemia cases. The kappa coefficient between BCHI and self-reported diabetes, hypertension, and hypercholesterolemia was 0.79, 0.59, and 0.49, respectively. Gender, age, and subjective health significantly affected the agreement in chronic condition classification between BHIS and BCHI.

**Conclusions:**

Data on reimbursed drugs is a potential alternative method in the surveillance of chronic diabetes. This procedure could be used in estimating disease prevalence but further validation is needed to evaluate its applicability and bias in other chronic conditions.

**Key messages:**

• BCHI is a possible alternative data source for the surveillance of diabetes in the population.

• BCHI overestimated hypertension and underestimated hypercholesterolemia prevalence.

